# No link between type I interferon autoantibody positivity and adverse reactions to COVID-19 vaccines

**DOI:** 10.1038/s41541-024-00829-9

**Published:** 2024-02-22

**Authors:** Ahmet Yalcinkaya, Marco Cavalli, Axel Cederholm, Maribel Aranda-Guillén, Anish Behere, Hedvig Mildner, Tadepally Lakshmikanth, Laura Gonzalez, Constantin Habimana Mugabo, Anette Johnsson, Olov Ekwall, Olle Kämpe, Sophie Bensing, Petter Brodin, Pär Hallberg, Mia Wadelius, Nils Landegren

**Affiliations:** 1 Department of Medical Biochemistry and Microbiology, Science for Life Laboratory, Uppsala University, Uppsala, Sweden; 2 Department of Medical Biochemistry, Hacettepe University Hospital, Ankara, Turkey; 3 Department of Immunology, Genetics and Pathology, Science for Life Laboratory, Uppsala University, Uppsala, Sweden; 4 Department of Medical Sciences, Clinical Pharmacogenomics, Science for Life Laboratory, Uppsala University, Uppsala, Sweden; 5 Center for Molecular Medicine, Department of Medicine (Solna), Karolinska Institutet, Stockholm, Sweden; 6 Department of Women’s and Children’s Health, Karolinska Institutet, Stockholm, Sweden; 7 Department of Pediatrics, Institute of Clinical Sciences, The Sahlgrenska Academy at University of Gothenburg, Gothenburg, Sweden; 8 Department of Rheumatology and Inflammation Research, Institute of Medicine, The Sahlgrenska Academy at University of Gothenburg, Gothenburg, Sweden; 9 Department of Clinical Science, University of Bergen, Bergen, Norway; 10 Department of Endocrinology, Karolinska University Hospital, Stockholm, Sweden; 11 Department of Molecular Medicine and Surgery, Karolinska Institutet, Stockholm, Sweden; 12 Department of Immunology & Inflammation, Imperial College London, London, UK

**Keywords:** Translational research, Vaccines, Adaptive immunity

## Abstract

Type I interferons act as gatekeepers against viral infection, and autoantibodies that neutralize these signaling molecules have been associated with COVID-19 severity and adverse reactions to the live-attenuated yellow fever vaccine. On this background, we sought to examine whether autoantibodies against type I interferons were associated with adverse events following COVID-19 vaccination. Our nationwide analysis suggests that type I interferon autoantibodies were not associated with adverse events after mRNA or viral-vector COVID-19 vaccines.

Vaccines for Coronavirus disease 2019 (COVID-19) have saved countless lives; however, it is evident that the general public has concerns due to their severe, albeit exceedingly rare, side effects^1^. Currently, there is limited evidence regarding factors underlying adverse events following immunization (AEFIs) with COVID-19 vaccines. These AEFIs include minor, self-contained events such as fatigue, fever, headaches, diarrhea etc., as well as severe complications such as thrombotic events, cardiac disease, various severe cytopenias, and neurological disorders^2^. Mechanistic explanations for some AEFIs do exist^3^, with particularly convincing results in certain complications, such as vaccine-induced immune thrombotic thrombocytopenia (VITT) that has been linked to autoantibodies against platelet factor 4-polyanion complexes^4^.

Being the first line of antiviral immunity, type I interferons (IFNs) have been rigorously explored for their roles in the pathogenesis and severity of COVID-19^5^. Research has shown that autoantibodies targeting type I IFNs predispose specific subsets of individuals to viral diseases, such as influenza pneumonia^6^ and West Nile encephalitis^7^, and also, these antibodies have been associated with severe disease and mortality in 5–10% of patients with COVID-19^8,9^. Since autoantibodies have been implicated in AEFIs caused by COVID-19 vaccines^4^ and considering that autoantibodies against type I IFNs have been associated with adverse reactions to the live-attenuated yellow fever vaccine^10^, it is conceivable that such autoantibodies may also contribute to AEFIs caused by COVID-19 vaccines.

The study included patients who had received at least one dose of the Comirnaty (Pfizer), Spikevax (Moderna) or Vaxzevria (AstraZeneca) vaccines and had been reported to the Swedish Medical Products Agency due to AEFIs that were attributed to these vaccines—based on World Health Organization criteria^11^. Ten AEFI subgroups were created (Supplementary Table 1). Blood samples were collected from April 2021 to March 2022 as part of the SWEDEGENE study (www.swedegene.se). Pre-pandemic blood donors (BD; *n* = 106) and patients with autoimmune polyendocrine syndrome type 1 (APS1; AIRE mutation-positive—high levels of type I IFN autoantibodies) were respectively included as the negative and positive control groups^12^. We screened for type I IFN autoantibodies, SARS-CoV-2 antibodies, and other technical/validatory targets, including anti-human IgG and Epstein-Barr virus nuclear antigen 1 (EBNA1), using a bead-based multiplex immunoassay and results were recorded in arbitrary units (AUs)^13^. Since multiplex assays can be impacted by non-specific binding or high background, which restrict the comparison of continuous data, we utilized a literature-defined cut-off to categorize patients, creating an ‘elevated response’ group including patients with reactivity against at least one IFN (>1500 AUs)^14^. Samples with elevated response were re-analyzed for the same IFNs with optimized ELISA protocols (IFNA2, A6, A8, and K).

A total of 290 patients, 163 (56.2%) females and 127 (43.8%) males, were enrolled. The most common AEFIs were coagulation-related (*n* = 100, 34.5%), followed by neurologic (*n* = 52, 17.9%), cardiac (*n* = 33, 11.4%), and allergic (*n* = 29, 10%) AEFIs. Patients with coagulation-related AEFI were significantly older and those with cardiac AEFI were significantly younger relative to other AEFI subgroups (Kruskal-Wallis *p* < 0.001, Eta^2: 0.226, dF: 10). All patients with allergic reactions were females and 86.2% had received the Comirnaty vaccine. Elapsed time from vaccination until AEFI onset was shortest in the allergic subgroup, and longest in those with cytopenic AEFIs (Kruskal-Wallis *p* < 0.001, Eta^2: 0.356, dF: 9) (Table 1).

**Table 1:** Blood donor and patient characteristics according to diagnosis category

	Count	Age	Sex, female	Vaccine			Time from vaccination to AEFI
	(n)	(years)	n (%)	Comirnaty	Spikevax	Vaxzevria	(days)
Blood donors	106	52.4 ± 12.6	55 (51.9%)	N/A	N/A	N/A	N/A
AEFI (overall)	290	56.5 ± 17.7	163 (56.2%)	173 (59.7%)	33 (11.4%)	84 (29%)	10.7 ± 11.6
Coagulation	100	64.2 ± 14^a^	54 (54%)	47 (47%)	9 (9%)	44 (44%)	13.33 ± 9.36
Neurologic	52	55.7 ± 17.3	26 (50%)	33 (63.5%)	7 (13.5%)	12 (23.1%)	10.38 ± 14.75
Allergic	29	45.4 ± 10.8	29 (100%)	25 (86.2%)	0 (0%)	4 (13.8%)	0.1 ± 0.27^c^
Cardiac	33	40.5 ± 18.9^b^	10 (30.3%)	21 (63.6%)	9 (27.3%)	3 (9.1%)	7.27 ± 7.89
Other	20	47.9 ± 15.8	15 (75%)	13 (65%)	2 (10%)	5 (25%)	8.61 ± 10.08
MACE	17	56.5 ± 18.2	5 (29.4%)	9 (52.9%)	2 (11.8%)	6 (35.3%)	11.88 ± 8.89
Cytopenia	17	58.5 ± 20	12 (70.6%)	10 (58.8%)	2 (11.8%)	5 (29.4%)	20.29 ± 8.3^d^
Systemic disease	10	69.4 ± 10.5	7 (70%)	7 (70%)	2 (20%)	1 (10%)	16.7 ± 24.2
Infection	9	67.8 ± 11	4 (44.4%)	6 (66.7%)	0 (0%)	3 (33.3%)	11.12 ± 10.49
Vascular	3	66.7 ± 6.1	1 (33.3%)	2 (66.7%)	0 (0%)	1 (33.3%)	4.33 ± 2.31

Groups are listed in rows to improve clarity.

Bonferroni-corrected *p* values have been reported below.

Continuous data summarized as mean ± standard deviation. Categorical data summarized with absolute (n) and relative frequency (%).

Comirnaty: Pfizer-BioNTech mRNA vaccine, Spikevax: Moderna mRNA vaccine, Vaxzevria: Oxford-AstraZeneca adenoviral vector vaccine.

*AEFI* adverse event following immunization, *N/A* not applicable, *MACE* major adverse cardiac event.

^a^Coagulation group significantly older compared to allergic (*p* < 0.001), blood donor (*p* < 0.001), cardiac (*p* < 0.001), and other (*p* = 0.002).

^b^Cardiac group significantly younger compared to coagulation (*p* < 0.001), systemic disease (*p* < 0.001), infection (*p* = 0.002), neurologic (*p* = 0.011), and cytopenia (*p* = 0.048).

^c^Time until AEFI significantly shorter in the allergic group compared to all other subgroups (*p* < 0.001, except for allergic vs. other and allergic vs. infection; *p* = 0.001 for both).

^d^Time until AEFI significantly longer in the cytopenia group compared to allergic (*p* < 0.001), neurologic (*p* = 0.001), cardiac (*p* = 0.002), and other (*p* = 0.018).

The detection of immunoglobulin G (IgG), EBNA1, and severe acute respiratory syndrome coronavirus 2 (SARS-CoV-2) antibodies largely yielded anticipated results: Anti-human IgG confirmed sample detection, anti-EBNA1 positivity was present at varying levels, and SARS-CoV-2 antibodies agreed with vaccination response in the AEFI group and were absent in the non-vaccinated BD and APS1 groups. A total of 51 patients appeared to have experienced SARS-CoV-2 (or other coronavirus) infection due to having positivity for nucleocapsid antibodies (>7 SD difference from BDs) which do not emerge as a result of vaccination. Medical histories revealed that possible COVID-19 infections were self-reported by 26 of these patients (8.9% of the whole population).

With respect to type I IFN antibodies, patients with APS1 showed strong immune reactivity towards almost all antigens; whereas, there were no strong reactivities and very few elevated responses (>1500 AUs) in the BD (*n* = 2) and AEFI (*n* = 9) groups (Fig. 1). The frequency of samples exhibiting elevated response to at least one IFN was similar in the AEFI and BD groups (3.1% vs. 1.9%, respectively, *p* = 0.734). Individuals with elevated response were similar to non-elevated subjects with respect to all characteristics (Table 2). ELISA confirmations yielded results similar to the bead-based assay for all antigens analyzed (Supplementary Fig. 1). There were four patients with equivocal results for IFNA2, A8 and K –necessitating analysis for neutralization properties. Inhibition of downstream signaling by samples was assessed with a type I IFN-stimulated reporter luciferase assay, revealing only one subject with neutralizing antibodies against IFNA2 (10 ng/ml) –an elderly individual with pulmonary emboli and deep venous thrombosis (Supplementary Fig. 2). Finally, dimensionality reduction performed on bead-based data also confirmed that results were unassociated with AEFI or AEFI subgroups (Supplementary Fig. 3).

**Fig. 1:**
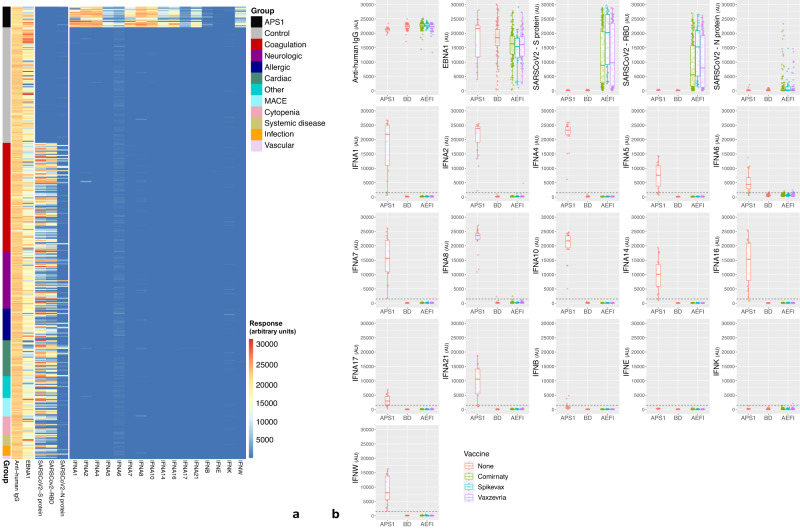
Heatmap and scatterplot visualization of autoantibodies against type I interferons in the study groups. Heatmap (**a**) illustrates autoantibody levels against type I interferons in all individuals. Color and color intensity show antibody response from the assay as described by the key. Clustering has been disabled for both rows and columns. The APS1 group shows elevated autoantibody levels for almost all examined antigens, barring IFNE and IFNK. This consistent increase in autoantibodies against multiple type I interferons is not observed in neither the BD nor the AEFI groups. Scatterplots (**b**) are used to compare antibody levels between the APS1 (*n* = 19), BD (*n* = 106) and AEFI (*n* = 290) groups, with respect to the type of vaccine for the AEFI group. The dashed lines in the scatterplots for interferons represent the threshold for ‘elevated response’ (1500 AUs). Boxplots were used to summarize data (median, 1st–3rd quartiles, whiskers represent 1.5X of interquartile range where applicable). The Y-axis has been kept constant throughout the scatterplot matrix to improve visibility and enable comparative assessment. When the three groups were compared in terms of antibody levels (Kruskal-Wallis test), it was found that the APS1 group had significantly higher values compared to the BD and AEFI groups for all analyses involving type I IFN antibodies (Bonferroni-corrected *p* = 0.001 for IFNK values in APS1 versus AEFI. Bonferroni-corrected *p* < 0.001 for all other pairwise comparisons). APS1 autoimmune polyendocrine syndrome type 1, BD blood donors, AEFI adverse events following immunization, AU arbitrary unit, IFN interferon, IgG immunoglobulin G, EBNA1 Epstein-Barr virus nuclear antigen 1, S protein spike protein, RBD receptor binding domain, N protein nucleocapsid protein, MACE major adverse cardiac event.

**Table 2:** Comparison of subjects with and without elevated response

	Elevated response for at least one IFN (>1500 AUs)		
	No	Yes	*p* value
Group, n (row %)			
APS1	0 (0%)	19 (100%)	BD vs. AEFI: 0.734^a^
BD	104 (98.1%)	2 (1.9%)	
AEFI	281 (96.9%)	9 (3.1%)	
Age, mean ± SD	55.4 ± 16.6	56 ± 14.8	0.970^b^
Sex, n (row %)			
Female	210 (96.3%)	8 (3.7%)	0.358^a^
Male	175 (98.3%)	3 (1.7%)	
Vaccine, n (row %)			
None (BDs)	104 (98.1%)	2 (1.9%)	0.057^a^
Comirnaty	170 (98.3%)	3 (1.7%)	
Spikevax	33 (100%)	0 (0%)	
Vaxzevria	78 (92.9%)	6 (7.1%)	
Vaccine dose, n (row %)			
None (BDs)	104 (98.1%)	2 (1.9%)	0.453^a^
Single dose	200 (96.2%)	8 (3.8%)	
Two doses	81 (98.8%)	1 (1.2%)	
Time from vaccination to AEFI (days), mean ± SD	10.9 ± 11.6	5.3 ± 9	0.051^b^
Diagnosis category in the AEFI group, n (row %)			
Coagulation	96 (96%)	4 (4%)	0.072^c^
Neurologic	52 (100%)	0 (0%)	
Allergic	25 (86.2%)	4 (13.8%)	
Cardiac	33 (100%)	0 (0%)	
Other	20 (100%)	0 (0%)	
MACE	16 (94.1%)	1 (5.9%)	
Cytopenia	17 (100%)	0 (0%)	
Systemic disease	10 (100%)	0 (0%)	
Infection	9 (100%)	0 (0%)	
Vascular	3 (100%)	0 (0%)	

*P* values have been calculated within the AEFI group (except for those concerning group, age, and sex).

*IFN* interferon, *AU* arbitrary unit, *APS1* autoimmune polyendocrine syndrome type 1, *BD* blood donor, *AEFI* adverse event following immunization, *MACE* major adverse cardiac event.

^a^Fisher’s exact test (2 × 2) or Freeman-Halton extension (2 × 3).

^b^Mann–Whitney U test.

^c^Likelihood-ratio test.

Since immunization remains the best and only reliable method to combat infectious diseases of viral origin, re-establishing and strengthening public trust in vaccines is crucial to prevent vaccine hesitancy^15^. For this purpose, it is imperative to accurately identify severe AEFIs and meticulously investigate the potential mechanisms at play. Our study shows that autoantibodies against type I IFNs are not found at an increased prevalence among individuals who suffered from AEFIs attributed to different mRNA and viral-vector COVID-19 vaccines. Type I IFN autoantibodies have been found in a high proportion of cases with adverse reactions to the live-attenuated yellow fever vaccine^10^. Taken together, these results suggest that impaired IFN response may be specifically associated with susceptibility to adverse reactions to live vaccines and not other types of vaccines.

Previous research reports various antibody-mediated mechanisms that potentially explain pathogenesis in certain adverse events, such as myocarditis^16^, thrombotic events^4^, Guillain-Barre syndrome^17^, transverse myelitis^18^ and allergic reactions^19^, which have been reviewed elsewhere^3^. Furthermore, a recent study emphasized that the presence of autoantibodies neutralizing type I IFNs could have implications on the utilization of mRNA vaccines in the population^20^. Perhaps the most notable complication associated with autoantibodies among vaccine recipients has been the description of VITT following administration of the Vaxzevria vaccine, which appeared to be caused by antibodies to platelet factor 4–polyanion complexes^4^. Further studies supported this finding^21^ and showed that VITT could manifest due to other vaccines^22^. Another finding worthy of mention is the demonstration of neutralizing anti-IL-1Ra autoantibodies in patients who developed myocarditis after vaccination^16^; however, more studies are required to assess this particular finding.

A limitation of the study includes the timing of blood sampling in relation to AEFI onset, and thus, differences in recruitment delay may be a cause of bias and could have influenced autoantibody levels in a few cases. Secondly, the presence of 26 patients with self-reports of potential COVID-19 infection could be perceived as a limiting factor; however, this limitation would have been confounding only if an increased prevalence of type I IFN autoantibodies had been found. Thirdly, the vaccines examined in this study were not live-attenuated vaccines, unlike the yellow fever vaccine—for which adverse events have been associated with autoantibodies to type I IFNs. Further studies must be performed in different populations where other COVID-19 vaccine types have been used. Nonetheless, the current study includes a large cohort of patients with AEFI in which neutralizing type I IFN autoantibodies were detected in only one subject. Additionally, patients with ‘elevated response’ (>1500 AUs) for certain isolated IFNs were similarly distributed in the BD and AEFI groups. Therefore, it is feasible to conclude that these autoantibodies are unlikely to be involved in the immunological mechanism(s) leading to AEFIs caused by mRNA or viral-vector COVID-19 vaccines.

## Methods

### Population and clinical data

Inclusion criteria were: having received at least one dose of a COVID-19 vaccine that was being administered in Sweden during the study period (Comirnaty, Spikevax, Vaxzevria), being diagnosed with a condition or event that was attributed to the vaccine, being 18 years or older at time of recruitment, and having the capability to provide informed consent. Causality assessment for AEFIs were performed according to WHO criteria, as described previously^11^. The vaccines are detailed as follows: Comirnaty is the proprietary name for the Pfizer-BioNTech mRNA vaccine, also known as BNT162b2. Spikevax is the proprietary name for the Moderna mRNA vaccine, also known as mRNA-1273. Vaxzevria is the proprietary name for the Oxford-AstraZeneca adenoviral-vector vaccine, also known as Covishield, ChAdOx1 nCoV-19, and AZD1222. Anonymous blood donor (BD) samples had been collected for research use before the COVID-19 pandemic at Uppsala University Hospital. APS1 patient samples were collected as part of an ongoing registry (Swedish Addison’s Disease Registry; ethics approval number: 2008/296-31/2).

Basic demographic data, elapsed time from vaccination until AEFI onset, and other clinical characteristics were collected from medical records and standardized questionnaires. Patients were classified according to AEFI diagnoses into the following groups: coagulation, neurological, allergic, cardiac, major adverse cardiac events (MACE), cytopenia, systemic disease, infection, vascular, and other. Exact diagnoses included in each group are detailed in Supplementary Table 1.

### Sample collection

Venous blood samples of patients with AEFIs (*n* = 290) were collected into EDTA-containing tubes. All samples were centrifuged to obtain plasma (1500 × *g*, 10 min, 4 °C), aliquoted, and sent for storage at –70 °C. De-identified samples were received at or transferred to the Medical Biochemistry and Microbiology Department of Uppsala University for analyses.

### Autoantibody screening via bead-based immunoassay

The screening of type I IFN autoantibodies was performed via an established bead-based anti-IgG assay that has been used previously with demonstration of reproducible results^13^. The large multiplex assay analysis plan was created to examine autoantibodies against 96 designated bead IDs (antigens, including technical controls) in a grand total of 2112 samples. Antibodies against IFN-α (IFNA1, 2, 4, 5, 6, 7, 8, 10, 14, 16, 17 and 21), IFN-β (IFNB), IFN-ɛ (IFNE), IFN-к (IFNK), and IFN-ω (IFNW) were measured in the study population for the specific purpose of the presented hypothesis. As part of the large multiplex assay, all samples underwent measurement of anti-human immunoglobulin G and antibodies against the primary proteins of severe acute respiratory syndrome coronavirus 2 (SARS-CoV-2), including Spike (S protein), receptor binding domain (RBD), and nucleocapsid (N protein). Antibody levels against the Epstein-Barr virus nuclear antigen 1 (EBNA1) were also measured to assess the detection reliability and reproducibility of measurements.

Samples (1 ul) were diluted with a 2-step process: 1:25 in phosphate-buffered saline (PBS) and then a further 1:10 in a solution containing 0.05% Tween-20, 3% BSA and 5% non-fat milk in PBS. Magnetic beads (MagPlex®, Luminex Corp.) were coupled with commercially-available type I IFNs (and other proteins examined) at a concentration of 3 ug per 1.5 × 10^6 beads. For coupling, the AnteoTech Activation Kit for Multiplex Microspheres was used (Catalog code: A-LMPAKMM-10). The diluted samples (250 ul total volume) were incubated with 5 μl of the bead solution for two hours at room temperature with slight agitation achieved by a shaker set to 650 rpm. Following the incubation, beads were magnetized before washing with 0.05% Tween-20 in PBS (3X), and then resuspended in 50 microliters of 0.2% paraformaldehyde for 10 min. After another 3X wash process, a 30-min incubation with the secondary antibody (Invitrogen, H10104 lot#2384336) was performed. Measurement was carried out with a FlexMap 3D instrument (Luminex Corp) and results were recorded in AUs.

### Autoantibody confirmation via ELISA

The initial multiplex screening (bead-based assay, Luminex) results were confirmed via optimized ELISA methods for several IFNs that were selected due to the presence of at least one sample with elevated response in the bead-based screening. That is, ELISA re-analysis was performed for a certain IFN if at least one ‘elevated response’ had been observed in either the AEFI or the BD group for said IFN (defined as >1500 AUs, based on Bastard et al.^14^). According to this criterion, we performed confirmatory testing for IFNA2 (number of samples with elevated response = 1), IFNA6 (*n* = 7), IFNA8 (*n* = 2), and IFNK (*n* = 1). For each antigen, the highest 8 samples (including those with elevated response) were included in the analyses (total *n* = 32). Starting sample dilution was 1:10 and was increased based on optimization goals described in the “Supplementary Methods” (1:20, 1:40, 1:80, 1:100, 1:160, 1:320, 1:1000, 1:2000, 1:5000, 1:10000, 1:20000, 1:25000, 1:50000, and 1:100000). In addition to the tested samples, we included three patients with APS1 as positive controls, one sample known to have high cross-reactivity (or non-specific binding), and three known-negative BDs during the course of each ELISA optimization (Supplementary Fig. 1).

### Neutralization analysis

The neutralization properties of equivocal responses (*n* = 4) detected in the multiplex autoantibody assay were analyzed via cell culture experiments –modified from previously-reported methods^23^. The experimental design involved (i) cell plating, (ii) co-transfection with *Firefly* (type I IFN-stimulable) and *Renilla* luciferase (constitutive expression) genes, (iii) stimulation with IFNA2 & addition of samples, and (iv) detection via a dual luciferase reporter assay. On day 1, HEK293T cells were seeded at 45000 cells/well in a 96-well plate (clear, flat-bottom, cell culture) with a final volume of 90 ul growth media per well (Gibco DMEM GlutaMAX + 10% fetal bovine serum + 100 units of penicillin-streptomycin). Transfection was performed with the *Firefly* pGL4.45[luc2P/ISRE/Hygro] and *Renilla* pRL-SV40 internal control luciferase vectors (Promega; #E414A and #E2231, respectively). The transfection solution was created in OptiMEM media with a 3:1 (ul:ug) ratio between the X-tremeGENE9 transfection reagent (Sigma-Aldrich; 6365787001) and total DNA (inter-vector ratio: 2:1 between *Firefly* and *Renilla*). The solution was incubated for 15 min and added to the wells (10 μl). On day 2, following overnight incubation, stimulation was performed with a final concentration of 10 ng/ml IFNA2 in wells (MedChemExpress; HY-P7022), except for non-stimulation controls. Immediately after stimulation, plasma samples were added into the wells to create a final plasma dilution of 1:10 in media, except for non-plasma controls. The plasma samples tested for neutralization included APS1 samples, AEFI samples with equivocal positivity (*n* = 4), and BD samples. On day 3, following 24 h of incubation, the Dual-Luciferase Reporter Assay System (Promega; #E1960) was used for analysis as described by the manufacturer (cell lysis, transfer of lysates to white opaque plates, and measurement with sequential addition of substrate and inhibitory/activating solutions). To perform quantification, we employed a plate reader that had luminescence quantification capabilities with magenta (*Firefly*) and green (*Renilla*) filters (Tecan, Magellan). The *Firefly:Renilla* ratio was used to assess neutralization. Technical controls confirmed experimental success, APS1 samples showed strong neutralization (ratios of <0.050), and BDs showed similar results to non-plasma controls–indicating non-neutralization (Supplementary Fig. 1).

### Statistics

To obtain descriptive data and perform statistical analyses, we utilized the SPSS software (version 25.0; IBM, NY, USA). Continuous data were summarized in the form of mean ± standard deviation. Categorical data were summarized with absolute (n) and relative frequency (%). Normality of distribution in continuous variables was checked via evaluation of Q-Q plots or histograms. When required, the lack of normal distribution was confirmed via the Shapiro-Wilk or the Kolmogorov-Smirnov (Lilliefors correction) tests. The Kruskal-Wallis test was used to compare continuous variables among diagnosis subgroups (and the BD group), and post hoc adjustments were performed with the Bonferroni correction. In the comparison of groups formed according to the presence/absence of ‘elevated response’ (>1500 AUs), analyses for continuous data were performed with the Mann–Whitney U test and we used appropriate Chi-square tests for categorical data. For data visualization in the form of scatterplots and the heatmap, we respectively used the “ggplot2” and “pheatmap” packages installed on RStudio software (“Cherry Blossom” release, 2023.03.1-Build 446)^24^. All code used to analyze data are available upon reasonable request from the corresponding authors. Dimensionality reduction was performed via principal component analysis (PCA) with use of the “prcomp” and “factoextra” packages in RStudio. The APS1 group was excluded from PCA. All type I IFN values were standardized with the calculation of Z-scores. Antibody levels for IgG, EBNA1 and SARS-CoV-2-related proteins were not included in the PCA in order to be able to detect the potential effects of IFNs –since smaller effects (and subgroups) could have been masked by variables with far greater impact (Supplementary Fig. 3).

### Reporting summary

Further information on research design is available in the Nature Research Reporting Summary linked to this article.

### Supplementary information

Supplementary text file

REPORTING SUMMARY

## Data Availability

The data that support the findings of this study and all code used for analyses are available from the corresponding authors upon reasonable request.
