# Supplementary material for: No link between type I interferon autoantibody positivity and adverse reactions to COVID-19 vaccines
Source: NPJ Vaccines. 2024 Feb 22;9:42. doi: 10.1038/s41541-024-00829-9 (PMC10883980; doi:10.1038/s41541-024-00829-9)
Supplement: Supplementary file 1 — Supplementary text file [file 41541_2024_829_MOESM1_ESM.pdf]

SUPPLEMENTARY FIGURES

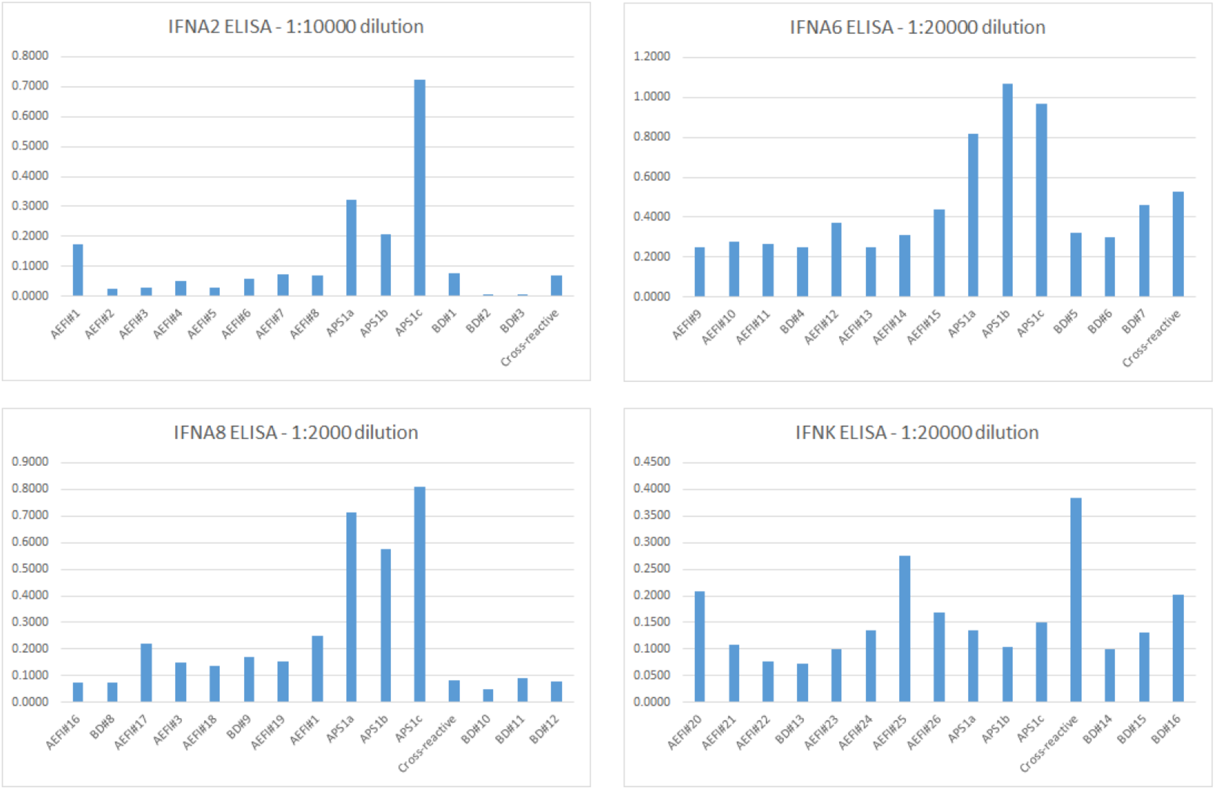

Supplementary Figure 1. Re-analysis of samples with elevated response via colorimetric ELISA

Confirmatory ELISA results for IFNA2 (1:10000 dilution), IFNA6 (1:20000 dilution), IFNA8 (1:2000 dilution) and IFNK (1:20000 dilution) using optimized protocols for each. In addition to tested samples (8 highest-response samples for each antigen), all runs included 3 patients with APS1, 3 known-negative BDs, and one sample with proven cross-reactivity against type I IFNs –for validation purposes. Please note that for IFNK, no elevated values were detected among APS1 patients in the bead-based assay; thus, the relatively-higher absorbance value for the cross-reactive sample is an expected result.

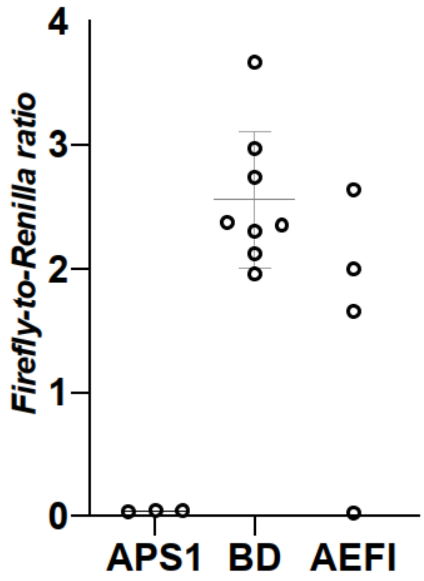

Supplementary Figure 2. Neutralization analysis results

The APS1 samples demonstrate complete inhibition (neutralization) of type I interferon stimulation. Similar result observed for one AEFI patient. BDs show no inhibition.

Abbreviations: APS1: autoimmune polyendocrine syndrome type 1, BD: blood donor, AEFI: adverse event following immunization.

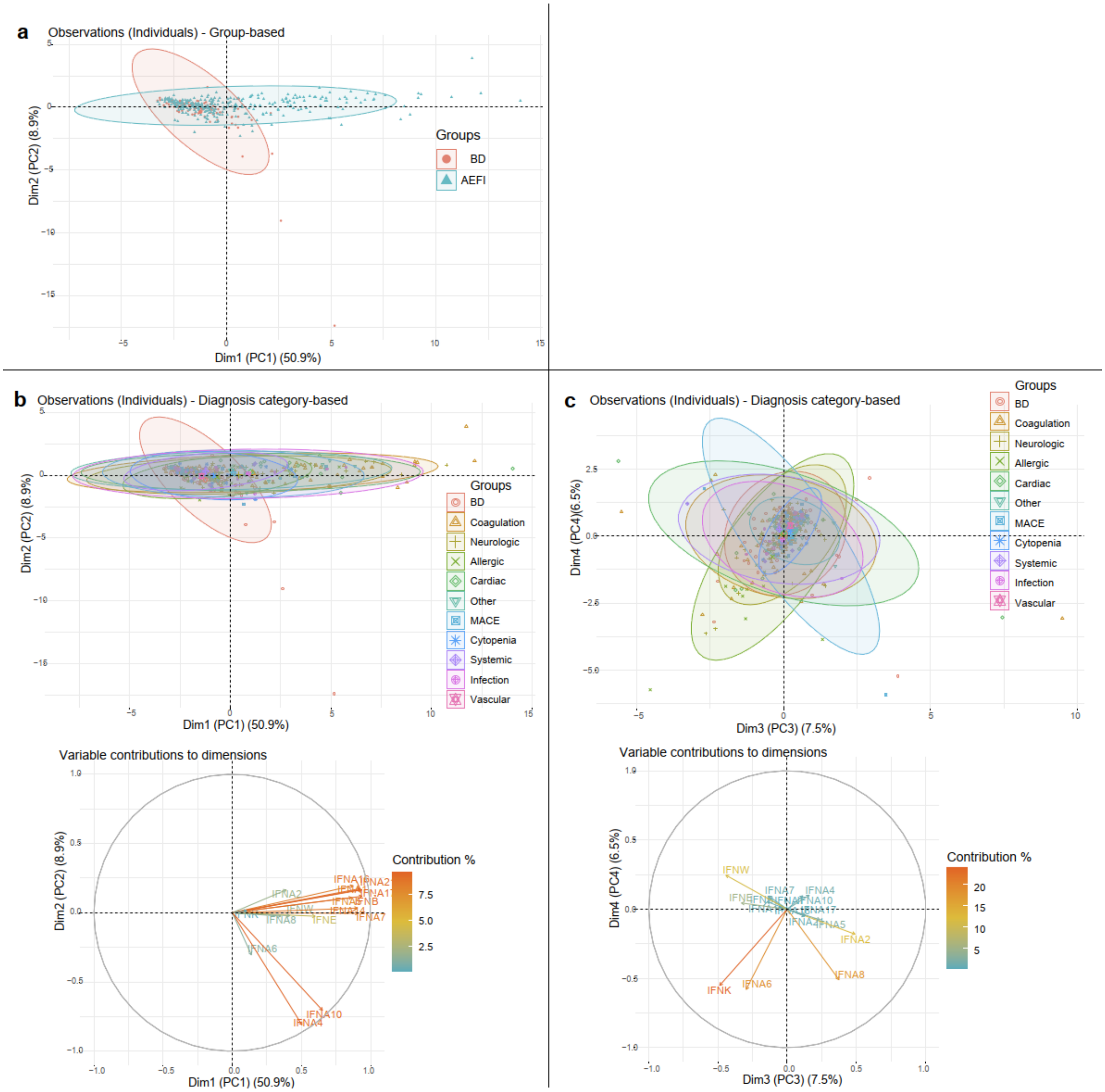

Supplementary Figure 3. Principle component analysis (observations and dimensions) for the AEFI vs. BD comparison and the AEFI subgroups vs. BD comparison

The PCA figures illustrate the plots of observations (individuals) based on PCs (dimensions) and show the explained variance for each dimension that had an Eigen value of >1.000 (PC1: 50.9%, PC2: 8.9%, PC3: 7.5%, and PC4: 6.5%). (a) Observations plotted on PC1 and PC2, with classification based on primary groups (AEFI vs. BD). (b) Observations plotted on PC1 and PC2 for AEFI subgroups vs. BD, and contributions to dimensions. (c) Observations plotted on PC3 and PC4 for AEFI subgroups vs. BD, and

contributions to dimensions. The results demonstrate that the dimensions created by PCA cannot be employed to distinguish between neither the AEFI vs. BD groups nor the AEFI subgroups.

Abbreviations: PCA: principal component analysis, PC1-2-3-4: principal component 1-2-3-4 (dimension 1-2-3-4), BD: blood donor, AEFI: adverse event following immunization, IFN: interferon, MACE: major adverse cardiac event.

SUPPLEMENTARY METHODS

ELISA protocol optimization and analysis

The final sample dilutions used for antibody detection via ELISA were optimized based on the targeted antigen with respect to aforementioned known controls (positive, negative, cross-reactive). For each ELISA optimization, we ensured that three criteria were met (optimization goals):

1) Detection of true antibodies (APS1, positive control).
2) Low response in negatives (similar to or slightly elevated compared to blanks).
3) The distinction of true positives from cross-reactive or non-specific-binding samples.

Below, we list the number of samples showing 'elevated response' (>1500 AUs) for each target, the number of samples included in the re-analysis, and the final sample dilution at which measurements were performed (based on optimization).

	**# of samples with >1500 AU**	**# of samples included (highest n)**	**Final optimized dilution used for analysis**
**IFNA1**	0	0	-
**IFNA2**	**1**	**8**	**1:10000**
**IFNA4**	0	0	-
**IFNA5**	0	0	-
**IFNA6**	**7**	**8**	**1:20000**
**IFNA7**	0	0	-
**IFNA8**	**2**	**8**	**1:1000–1:2000**
**IFNA10**	0	0	-
**IFNA14**	0	0	-
**IFNA16**	0	0	-
**IFNA17**	0	0	-
**IFNA21**	0	0	-
**IFNB**	0	0	-
**IFNE**	0	0	-
**IFNK**	**1**	**8**	**1:10000-1:20000**
**IFNW**	0	0	-

The general ELISA protocol was as follows:

Preparation:

1) Sample dilution buffer: phosphate-buffered saline (PBS). Two PBS tablets for 1000 ml ddH2O (UPW), wait for mix.
2) Wash buffer: PBST (0.1%): Measure 500 ml of sample dilution buffer (PBS) and transfer to new bottle. Add 0.1% Tween-20 (500 ul), pipette very slowly, wait for mix.
3) Blocking buffer: 3% BSA in 0.05% PBST. Measure 50 ml of wash buffer and transfer to new bottle. Add 50 ml of PBS and 3 gr of BSA, wait for mix.
4) Secondary antibody dilution buffer: 0.1% BSA in 0.02% PBST. Measure 20 ml of wash buffer, mix with 80 ml of PBS in new bottle (final: 100 ml). Add 0.1 gr of BSA, wait for mix.

Procedure for high-binding, half-area, clear 96-well plates:

1) Coat with 1-4 ug/ml (40 ul volume) target protein, overnight @4°C.
2) Next morning, wash 3X with 100 ul wash buffer for each well in each wash.
3) Block with 100 ul of blocking buffer, 90 minutes @RT.
4) Wash 5X with 100 ul wash buffer.
5) Incubate with 40 ul samples in duplicate, 45 minutes @RT.
    a. 1:10–1:100000-diluted AEFI samples
    b. 1:10–1:100000-diluted control samples (positive, negative and cross-reactive)
5) Wash 5X with 100 ul wash buffer.
6) Incubate with 50 ul detection antibody, 45 minutes @RT.
    a. 1:10000-diluted anti-IgG/A/M or anti-IgG (both HRP-conjugated).
    b. Dilution performed with the secondary antibody dilution buffer.
7) Wash 5X with 100 ul wash buffer.
8) Incubate with 50 ul TMB (HRP substrate), 5-15 minutes.
9) Add 40 ul stop solution (0.2 M H_2SO_4).
10) Measure at 450 nm.

Principle components analysis

PCA plots were created for dimensions with Eigen values greater than 1.000 (PCs 1 vs. 2 and PCs 3 vs. 4).

The principal components (dimensions) obtained from PCA are listed below.

	Eigen value	Variance %	Cumulative variance %
**Dimension 1**	**8.139**	50.868	50.869
**Dimension 2**	**1.425**	8.907	59.776
**Dimension 3**	**1.199**	7.496	67.253
**Dimension 4**	**1.047**	6.541	73.814
Dimension 5	0.956	5.978	79.791
Dimension 6	0.784	4.899	84.690
Dimension 7	0.767	4.796	89.487
Dimension 8	0.619	3.867	93.354
Dimension 9	0.419	2.618	95.972
Dimension 10	0.195	1.220	97.192

Dimension 11	0.173	1.083	98.275
Dimension 12	0.104	0.649	98.924
Dimension 13	0.075	0.466	99.390
Dimension 14	0.053	0.334	99.724
Dimension 15	0.029	0.183	99.907
Dimension 16	0.015	0.093	100.000

SUPPLEMENTARY TABLE

Supplementary Table 1. Diagnosis categories and the exact diagnoses included in the major groups.

Coagulation	Neurologic	Allergic	Cardiac	Other	MACE	Cytopenia	Systemic disease	Infection	Vascular
Amaurosis fugax	Abducens palsy	Allergic reaction	Myocarditis	Acute macular neuroretinopathy	Transient ischemic attack (TIA)	Agranulocytosis	Amyopathic dermatomyositis	Shingles	Capillary leak syndrome
Arterial thrombosis	Anosmia	Anaphylactic shock	Pericarditis	Acute tubular necrosis	Cerebral infarction	Hemolytic anemia	Multisystem inflammatory syndrome in adults (MIS-A)	Herpes zoster ophthalmicus	Vasculitis
Hematoma	Numbness	Anaphylactic reaction	Perimyocarditis	Alveolitis	Myocardial infarction	Pancytopenia	Polymyalgia rheumatica		
Bleeding	Encephalitis	Angioedema	Supraventricular tachycardia (extra stroke)	Aortic dissection		Thrombocytopenia	Rheumatoid arthritis (RA)		
Cerebral sinus thrombosis	Encephalomyelitis	Tingling sensation	Takotsubo cardiomyopathy	Connective tissue disease			Still's disease		
Deep venous thrombosis (DVT)	Facial palsy	Urticaria		Bullous pemphigoid			Temporal arteritis		
Ecchymosis	Guillain-Barré syndrome			Cryptogenic organizing pneumonia (COP)					
Epistaxis	Hearing loss			Erythema multiforme					
Hemoptysis	Sensory disturbance			Exanthema maculopapular (rash)					
Pulmonary embolism	Smell disorder (parosmia)			Flushing					
Mesenteric vein thrombosis	Meningitis			Glossitis					
Petechiae	Myelitis			Hematuria					
Retinal hemorrhage	Myelopathy			Hemolytic anemia					
Retinal artery occlusion	Neuropathy			Hyperthyroidism					
Retinal artery thrombosis	Paresthesia			IgA nephritis					
Retinal vein thrombosis	Polyneuropathy			Pneumonitis					
Sinus thrombosis	Taste disorder (dysgeusia)			Interstitial lung disease					
Subarachnoid hemorrhage	Vocal cord paralysis			Carotid artery dissection					
Thrombophlebitis	Trochlear nerve paralysis			Elevated liver enzymes					
Thrombosis	Vestibular neuritis			Pulmonary effects					
Venous thrombosis	Dizziness			Splenic infarction					
				Myalgia					
				Neuromyelitis optica					
				Omentum infarction					
				Optic infarction					
				Pancreatitis					
				Pemphigoid					
				Pityriasis rubra pilaris					
				Pleuritis					
				Progressive external ophthalmoplegia					
				Rhabdomyolysis					
				Scleroderma					
				Sweet's syndrome					
				Thyroiditis					
