# Supplementary material for: No link between type I interferon autoantibody positivity and adverse reactions to COVID-19 vaccines
Source: NPJ Vaccines. 2024 Feb 22;9:42. doi: 10.1038/s41541-024-00829-9 (PMC10883980; doi:10.1038/s41541-024-00829-9)
Supplement: Supplementary file 2 — REPORTING SUMMARY [file 41541_2024_829_MOESM2_ESM.pdf]

nature portfolio

Corresponding author(s): Ahmet Yalcinkaya, Nils Landegren

Last updated by author(s): Oct 6, 2023

Reporting Summary

Nature Portfolio wishes to improve the reproducibility of the work that we publish. This form provides structure for consistency and transparency in reporting. For further information on Nature Portfolio policies, see our Editorial Policies and the Editorial Policy Checklist.

Statistics

For all statistical analyses, confirm that the following items are present in the figure legend, table legend, main text, or Methods section.

n/a	Confirmed	
☐	☒	The exact sample size (*n*) for each experimental group/condition, given as a discrete number and unit of measurement
☐	☒	A statement on whether measurements were taken from distinct samples or whether the same sample was measured repeatedly
☐	☒	The statistical test(s) used AND whether they are one- or two-sided<br>*Only common tests should be described solely by name; describe more complex techniques in the Methods section.*
☒	☐	A description of all covariates tested
☐	☒	A description of any assumptions or corrections, such as tests of normality and adjustment for multiple comparisons
☐	☒	A full description of the statistical parameters including central tendency (e.g. means) or other basic estimates (e.g. regression coefficient) AND variation (e.g. standard deviation) or associated estimates of uncertainty (e.g. confidence intervals)
☐	☒	For null hypothesis testing, the test statistic (e.g. *F*, *t*, *r*) with confidence intervals, effect sizes, degrees of freedom and *P* value noted<br>*Give P values as exact values whenever suitable.*
☒	☐	For Bayesian analysis, information on the choice of priors and Markov chain Monte Carlo settings
☒	☐	For hierarchical and complex designs, identification of the appropriate level for tests and full reporting of outcomes
☒	☐	Estimates of effect sizes (e.g. Cohen's *d*, Pearson's *r*), indicating how they were calculated

Our web collection on statistics for biologists contains articles on many of the points above.

Software and code

Policy information about availability of computer code

Data collection	RStudio “Cherry Blossom” release, 2023.03.1-Build 446<br>SPSS v25<br>GraphPad Prism 9
Data analysis	RStudio “Cherry Blossom” release, 2023.03.1-Build 446<br>SPSS v25

For manuscripts utilizing custom algorithms or software that are central to the research but not yet described in published literature, software must be made available to editors and reviewers. We strongly encourage code deposition in a community repository (e.g. GitHub). See the Nature Portfolio guidelines for submitting code & software for further information.

Data

Policy information about availability of data

All manuscripts must include a data availability statement. This statement should provide the following information, where applicable:

- Accession codes, unique identifiers, or web links for publicly available datasets
- A description of any restrictions on data availability
- For clinical datasets or third party data, please ensure that the statement adheres to our policy

The data that support the findings of this study are available from the corresponding authors upon reasonable request.

Research involving human participants, their data, or biological material

Policy information about studies with human participants or human data. See also policy information about sex, gender (identity/presentation), and sexual orientation and race, ethnicity and racism.

Reporting on sex and gender	This study is a part of a population-based project that collects data from all individuals with AEFI. It will collect data based on the presence of adverse events, regardless of the sex or gender of the individuals. The hypotheses do not consider sex or gender as a parameter for inclusion or exclusion, as this would bias the results. However, the outcomes are ultimately examined in this regard. For instance, all patients who suffered from allergic AEFIs were females, which has been emphasized in the paper. With respect to the definitions in this manuscript, sex was used to describe biological sex only and none of the analyses have any relevance or reference to gender as a social construct.
Reporting on race, ethnicity, or other socially relevant groupings	No such categorization was used.
Population characteristics	Age, sex, vaccination data (type/brand and dose), diagnoses. and antibody levels against SARS-CoV-2 proteins and EBNA1 were measured. Since the study aimed to assess the prevalence of type I IFN autoantibodies in our cohort, these 'potential covariates' would have no bearing on the outcomes.
Recruitment	From the manuscript: "A limitation of the study includes the timing of blood sampling in relation to AEFI onset, and thus, differences in recruitment delay may be a cause of bias and could have influenced autoantibody levels in a few cases." No other biases would be relevant to note for such a straightforward research; however, it is possible to mention that the diagnoses were made by different physicians employed by different institutions throughout the country. This could have led to a selection bias (and possible ascertainment bias due to different interpretations of severity); however, we can expect that the causality assessment would have prevented any strong variations in the definition/diagnosis of AEFIs and their severity.
Ethics oversight	Swedish Ethics Review Authority #2021-06262-01

Note that full information on the approval of the study protocol must also be provided in the manuscript.

Field-specific reporting

Please select the one below that is the best fit for your research. If you are not sure, read the appropriate sections before making your selection.

☒ Life sciences ☐ Behavioural & social sciences ☐ Ecological, evolutionary & environmental sciences

For a reference copy of the document with all sections, see nature.com/documents/nr-reporting-summary-flat.pdf

Life sciences study design

All studies must disclose on these points even when the disclosure is negative.

Sample size	This is the first report from an overarching project examining AEFIs and has recruited patients from the whole of Sweden. The sampling and analyses are ongoing. There is a paucity of tangible data concerning the frequency of type I IFN autoantibodies among patients who developed AEFI following receipt of mRNA or viral-vector vaccines --making data-driven power analyses impossible. Nonetheless, based on a 1% frequency of autoantibodies in the population (age-corrected estimate) and a 4% frequency as the upper threshold to accept H0 (based on lower thresholds of autoantibody positivity in patients with severe COVID-19), we can calculate the population size required to detect a significant difference at an alpha error of 5% and power values of 80% and 95% (1-beta). Using the Binomial, one-sample Proportion Test via G*Power 3.1.9.7, we can see that the required sample size is at minimum 137 subjects (80% power) and at maximum 261 subjects (95% power). Therefore, the present study has sufficient power to reject H0 based on background data and aforementioned assumptions.
Data exclusions	No data-points were excluded; however, patients were excluded based on causality assessment, which has been described and referenced in the manuscript.
Replication	No replication studies were performed. However, analyses were performed with duplicates for each methodology used. We have no reason to suspect that the results would not be reproducible.
Randomization	Since the aim of the study was to examine whether type I IFN autoantibodies were present in the population, the covariates would be expected to have minimal impact on the results. The patients examined in this study were from a nationwide cohort and patients were only excluded based on causality assessment. Furthermore, no experimental groups were formed except for categorization purposes, and therefore, randomization is of no concern for the present study.
Blinding	During data collection, samples were received with minimal identifying information. The primary laboratory analysis (multiplex assay for autoantibodes) was performed by a separate group of researchers before receipt of clinical information (diagnosis, age, vaccine etc.); thus, the experimentation was performed in a blinded fashion. Data analysis was not blinded as the dataset included all available information; however, it should be re-iterated that the present study reports negative results.

Reporting for specific materials, systems and methods

We require information from authors about some types of materials, experimental systems and methods used in many studies. Here, indicate whether each material, system or method listed is relevant to your study. If you are not sure if a list item applies to your research, read the appropriate section before selecting a response.

Materials & experimental systems

n/a	Involved in the study
☐	☒ Antibodies
☐	☒ Eukaryotic cell lines
☒	☐ Palaeontology and archaeology
☒	☐ Animals and other organisms
☒	☐ Clinical data
☒	☐ Dual use research of concern
☒	☐ Plants

Methods

n/a	Involved in the study
☒	☐ ChIP-seq
☒	☐ Flow cytometry
☒	☐ MRI-based neuroimaging

Antibodies

Antibodies used	Secondary antibody used for multiplex assay:<br>F(ab')2-Goat anti-Human IgG Fc Secondary Antibody, PE-conjugated<br>Invitrogen #H10104, lot #2384336
Validation	CoA available for lot #2384336, signed on December 9, 2021

Eukaryotic cell lines

Policy information about cell lines and Sex and Gender in Research

Cell line source(s)	HEK293T cells, obtained by a colleague from American Type Culture Collection (ATCC).
Authentication	Contaminations have been checked regularly as part of the routine processes of a cell culture lab.
Mycoplasma contamination	Contaminations have been checked regularly as part of the routine processes of a cell culture lab. Cells were used only for neutralization analyses and were received from a colleague. Mycoplasma contamination was not checked during the 7 passages that the cells were used for neutralization assays.
Commonly misidentified lines<br>(See ICLAC register)	does not apply

Plants

Seed stocks	does not apply (please be aware that there is a problem with the PDF, the check-box above does not remove this section)
Novel plant genotypes	does not apply (please be aware that there is a problem with the PDF, the check-box above does not remove this section)
Authentication	does not apply (please be aware that there is a problem with the PDF, the check-box above does not remove this section)
